# Brain metastasis development and poor survival associated with carcinoembryonic antigen (CEA) level in advanced non-small cell lung cancer: a prospective analysis

**DOI:** 10.1186/1471-2407-9-119

**Published:** 2009-04-22

**Authors:** Oscar Arrieta, David Saavedra-Perez, Roberto Kuri, Alejandro Aviles-Salas, Luis Martinez, Daniel Mendoza-Posada, Patricia Castillo, Alma Astorga, Enrique Guzman, Jaime De la Garza

**Affiliations:** 1 Department of Medical Oncology, Instituto Nacional de Cancerologia, Mexico City, Mexico; 2 Department of Pathology, Instituto Nacional de Cancerologia, Mexico City, Mexico; 3 Department of Clinical Research, Instituto Nacional de Cancerologia, Mexico City, Mexico; 4 Clinic of Lung Cancer and Thoracic Tumors, Instituto Nacional de Enfermedades Respiratorias, Mexico City, Mexico

## Abstract

**Background:**

Central nervous system is a common site of metastasis in NSCLC and confers worse prognosis and quality of life. The aim of this prospective study was to evaluate the prognostic significance of clinical-pathological factors (CPF), serum CEA levels, and EGFR and HER2 tissue-expression in brain metastasis (BM) and overall survival (OS) in patients with advanced NSCLC.

**Methods:**

In a prospective manner, we studied 293 patients with NSCLC in IIIB-IV clinical stage. They received standard chemotherapy. CEA was measured prior to treatment; EGFR and HER2 were evaluated by immunohistochemistry. BM development was confirmed by MRI in symptomatic patients.

**Results:**

BM developed in 27, and 32% of patients at 1 and 2 years of diagnosis with adenocarcinoma (RR 5.2; 95% CI, 1.002–29; p = 0.05) and CEA ≥ 40 ng/mL (RR 11.4; 95% CI, 1.7–74; *p *< 0.01) as independent associated factors. EGFR and HER2 were not statistically significant. Masculine gender (RR 1.4; 95% CI, 1.002–1.9; *p *= 0.048), poor performance status (RR 1.8; 95% CI, 1.5–2.3; *p *= 0.002), advanced clinical stage (RR 1.44; 95% CI, 1.02–2; *p *= 0.04), CEA ≥ 40 ng/mL (RR 1.5; 95% CI, 1.09–2.2; *p *= 0.014) and EGFR expression (RR 1.6; 95% CI, 1.4–1.9; *p *= 0.012) were independent associated factors to worse OS.

**Conclusion:**

High CEA serum level is a risk factor for BM development and is associated with poor prognosis in patients with advanced NSCLC. Surface expression of CEA in tumor cells could be the physiopathological mechanism for invasion to CNS.

## Background

Lung cancer is the first cause of cancer death in the world. Eighty five percent of patients are diagnosed yearly with non-small cell lung cancer (NSCLC). Despite efforts, innovations, and progress in diagnosis and treatment of these patients, overall survival (OS) at 5 years of diagnosis is only 15% [1].

The central nervous system (CNS) is a devastating and frequent site of metastasis development in NSCLC. The reported incidence of CNS metastasis in patients with NSCLC is 54% [2] with an OS of <1 year after diagnosis [3-5]. Age [3], clinical stage [6], gender [7], and initial treatment period [8] are some of the reported with CNS metastasis development-related factors in patients with NSCLC; however, due to their lack of specificity, we are required to detect biomarkers to predict brain metastasis in patients with NSCLC.

Carcinoembryonic antigen (CEA, CEA-related cell adhesion molecule 5, CEACAM5) is an oncofetal protein attached to epithelial-cell apical membrane via its c-terminal glycosylphosphatidylinositol anchor, a member of the immunoglobulin superfamily of cell adhesion molecules (IgCAMs) [9]. CEA is usually over-expressed in a variety of neoplasms, such as colorectal, breast, bladder, gastric, pancreatic, and lung carcinomas [10]. CEA protein levels were found to correlate with its mRNA levels in cells and tissues examined, suggesting that CEA overexpression in cancer cells involves CEA-gene transcriptional activation [9]. CEA-related cell adhesion molecules (CAMs) are involved in cell-cell recognition and modulate cellular processes [11]. High serum CEA levels have been associated with advanced disease and tumor relapse in resected NSCLC [12-19]. Despite this widely reported information, there are no studies on serum CEA levels in advanced NSCLC and brain metastasis development.

The family of epidermal growth factor receptors (EGFRs) plays an important role in proliferation and cell survival. It is composed of the following four different receptors: 1) epidermal growth factor receptor (EGFR, erbb1); 2) HER2/neu (erbb2); 3) HER3 (erbb3), and 4) HER4 (erbb4). In NSCLC, reported incidence of EGFR tissue expression is 43–89% [20] and it has been associated with a worse prognosis [21]; nonetheless, evidence concerning its role as a prognostic factor remains controversial [22-26]. With an incidence of 25% in breast cancer, HER2 tissue expression is associated with poor disease-free survival and OS compared with patients who are HER2-negative [27]. Recent reports correlate this with brain metastasis development [28]. In patients with NSCLC, HER2 tissue expression entertains an incidence of 11–32% [29] and has been linked with worse OS at 3 and 5 years of diagnosis [30].

The objective of this study was to evaluate prospectively manner the prognostic significance of clinical-pathological factors, serum CEA levels, and EGFR and HER2 lung expression in brain metastasis development and OS in patients with recent diagnosis of advanced NSCLC (clinical stage IIIB-IV) treated with platin-based cytotoxic chemotherapy or EGFR inhibitors.

## Methods

### Patients

Between March 2005 and June 2007, patients with recent histological diagnosis of advanced NSCLC without previous treatment and referred to the Lung cancer and Thoracic Tumors Clinic (Instituto Nacional de Cancerología and Instituto Nacional de Enfermedades Respiratorias, Mexico City, Mexico) were enrolled in this prospective study. This work was approved by bioethical and research committees of each institution, and patient informed consent was obtained before enrollment. Histological diagnosis of primary NSCLC was established according to the revised classification of lung tumors of World Health Organization and the International Association for Lung Cancer Study. During pre-treatment clinical evaluation, we focused on physical examination, weigh loss, and performance status (according to the Eastern Cooperative Oncologic Group scale, ECOG). Thoracic, upper abdominal computed tomography (CT) and bone scintillography were used in disease staging. Only patients with IIIB (T4 N0-1-2 M0 or any T N3 M0: tumor of any size that invades mediastinum, heart, great vessels trachea, esophagus, vertebral body, carina; or tumor with a malignant pleural or pericardial effusion, or with satellite tumor nodule(s) within the ipsilateral primary-tumor lobe of the lung [T4], no regional lymph node metastasis [N0], metastasis to ipsilateral peribronchial and/or ipsilateral hiliar lymph nodes, and intrapulmonary lymph nodes involved by direct extension of the primary tumor [N1], metastasis to ipsilateral mediastinal and/or subcarinal lymph node(s) [N2], or metastasis to contralateral mediastinal, contralateral hiliar, ipsilateral or contralateral scalene, or supraclavicular lymph node(s) [N3], and no distant metastasis [M0]) and IV clinical stage (any T any N M1: distant metastasis present) were enrolled [31,32]. Chemotherapy was based on platin or EGFR inhibitors. Six different schedules were instituted, including gemcitabine, vinorelbine, or paclitaxel combined with cisplatin or carboplatin and erlotinib.

### Samples

Analyzed variables comprised smoking history, gender, general condition, histology, brain metastasis development, serum CEA levels, and EGFR and HER2 tissue expression.

CEA levels in serum were evaluated prior to chemotherapy. CEA was measured by solid-phase, two-site sequential chemiluminescent immunometric assay (IMMULITE 2000-CEA Analyzer, Siemens, Inc., Los Angeles, CA) with an analytical sensibility of 0.15 ng/mL and a high-dose Hook effect at levels of >250,000 ng/mL. Serum samples (15 μL serum) were collected using the same method for each patient and stored if necessary at -20°C before processing. Cut-off points for resuming tables were selected according to the previously reported normal value for CEA serum level (< 10 ng/mL) [10].

EGFR and HER2 tissue expression were evaluated by immunohistochemistry (IHC). IHC was performed on formalin-fixed, paraffin-embedded tissue sections from primary lung tumors. 5 μm sections were placed on chemically charged slides. Sections were deparaffinized and rehydrated in a series of alcohols and xylene according to established procedures. Following deparaffination, sections were immersed in an antigen-retrieval solution (Dako Corporation) for 40 min at 95°C. Endogen peroxidase was blocked with 3% H_2_O_2 _in absolute methanol for 5 min. Slides were incubated with rabbit antibody human HER2 (Hercep Test, Dako Corporation) for 30 min, and staining was completed using Dako Rabbit Evision Plus Kit (Dako Corporation). The antibody binding site was visualized using diaminobenzidine reagent for 5 min. The slides were counterstained with Mayer's hematoxylin. Immunostainig for EGFR was accomplished using the monoclonal mouse antibody to EGFR (1:100, Zymed Laboratories, San Francisco, CA). The slides were incubated with peroxide block for 10 min, this followed by incubation for 10 min with proteinase K. After incubation of slides with primary antibody for 30 min, staining was completed by incubation with monoclonal-labeled polymer for 10 min. The antibody binding site was visualized using diaminobenzidine reagent. Finally, the slides were counterstained with Mayer's hematoxylin.

EGFR and HER2 were examined using light microscopy. For EGFR, we considered negative tissue expression if the stain was heterogeneous in ≤ 25% of the sample, and positive tissue expression if stain was homogeneous >25% of sample. For semi-quantitative evaluation of HER2, all slides were scored following the guidelines for scoring HercepTest: 0, completely negative or membrane staining in fewer than 10% of tumor cells; 1+, faint membranous staining in >10% of the tumor cells; 2+, weak or moderate complete staining in >10% of tumor cells, and 3+, strong complete membrane staining in >10% of the tumor cells. IHC was graded by a single pathologist (AAS), who was blinded to clinical characteristics and outcomes.

### Follow-up

In presence of neurological symptoms (persistent headache, neurological focalization, motor deficits, or abnormal behaviour), brain magnetic resonance imaging (MRI) was performed. Monitoring of disease (including primary endpoints, e.g., OS and brain metastasis development) was carried out by means of clinical follow-up.

### Statistical analysis

With a descriptive purpose, we resumed each continuous variable as arithmetic mean, median and standard deviation (SD), and categorical variables as proportion with 95% confidence interval (95% CI). For inferential comparisons, we employed Student *t *or Mann-Whitney *U *test, according to data distribution (normal or non-normal, determined with Kolmogorov-Smirnov test). To calculate statistical significance between categorical variables, we used chi-square or Fisher exact test. Statistically significant and borderline results (*p *< 0.1) were included in multivariate logistic regression analysis. CNS metastasis development and OS were defined as the period from date of histological diagnosis to date of confirmed diagnosis of brain metastasis by MRI and to date of death, respectively; both were analyzed with Kaplan-Meier method, and sub-groups were compared with log-rank test. To analyze survival curves, each variable was dichotomized. HER2 tissue expression was considered negative if IHC was classified as 0 or +1, and positive if classified as +2 and +3. For EGFR, tissue expression was negative or positive if IHC was positive in <25% or ≥ 25% of tumor cells, respectively. Statistically significant or borderline (*p *< 0.1) variables on univariate analysis were included in multivariate analysis utilizing Cox proportional hazards model. Statistical significance was determined with a *p *< 0.05 in a two-sided test. SPSS software package (version 14.0; SPSS, Inc., Chicago, IL) was employed for data analysis.

## Results

### Patients and samples

Two hundred ninety three consecutive patients were prospectively studied. Patient characteristics are shown in Table 1. Mean for age was 60.7 ± 0.7 years. Only 54% of patients had smoking history. The majority of tumors were adenocarcinoma (65%) classified as moderate or high histological grade (36 and 54%, respectively). Seventy one percent of patients were diagnosed in a metastatic stage, and 29% were in IIIB clinical stage. Of patients with metastasis at time of diagnosis, 18.6% had CNS metastasis and 7.5%, liver metastasis. Most patients were referred to our Institute without paraffin blocks and in others the diagnosis was based on cytopathological samples; even, when tissue samples were available, some of them were insufficient to perform the immunohistochemistry. Because of these reasons, we only analyzed 85 biopsies of primary tumor to determine tissue expression of EGFRs types 1 and 2, of which, 59% were positive for EGFR and only 7% for HER2 tissue expression.

**Table 1:** Baseline patient characteristics

N = 293	Mean ± SE	Patients (%)
**Age **(years)	60.7 ± 0.7	
**Gender**		
Female		44
Male		56
**Smoking History**		
Positive		53.9
Negative		46.1
**ECOG**		
1		45.4
2		23.5
3		31.1
**Clinical Stage**		
III B		29.4
IV		70.6
**Histology**		
Adenocarcinoma		64.8
*Other		35.2
**Histological Grade**		
Low		10.3
Moderate		35.9
High		53.8
**Metastasis at Diagnosis**		
CNS		18.6
Liver		7.5
**Other		44.9
**First Line Treatment**		
Chemotherapy		87.4
Tyrosine-kinase inhibitors		12.6
EGFR***		
Positive		58.6
Negative		41.4
HER2***		
Positive		6.8
Negative		93.2

SE: Standard Error.

ECOG: Eastern Oncology Cooperative Group Scale.

*Other: Squamous, Giant Cells, Undifferentiated.

CNS: Central Nervous System.

**Other: Lymph Nodes, Bones, Contralateral Lung.

EGFR: Epidermal Growth-Factor Receptor.

***: Tissue expression was determined only in 85 different

primary tumor samples (see text).

### Serum levels of CEA

In 42.8, 32.3, 22.2, and 21.4% of patients, basal serum CEA level was ≥ 10, 20, 40, and 50 ng/mL at diagnosis, respectively (median ± standard deviation, 6.3 ± 1,021 ng/mL; range, 0.2–15,475 ng/mL). Age, gender, positive smoking history, status performance, histological-grade of differentiation and presence of liver metastasis at diagnosis were not associated to basal CEA serum levels ≥ 40 ng/mL. However, at the bi- and multivariate analysis, factors associated with basal CEA serum levels ≥ 40 ng/mL were adenocarcinoma histological type (frequency of 41.3% compared with 14.1% of patients with squamous or large-cell histologies, RR 1.6; 95% CI, 1.4–3.4; *p *= 0.005) and presence of CNS metastasis at diagnosis (frequency of 77.7% compared with 12.3% of patients without CNS metastasis at diagnosis, RR 14.05; 95% CI, 5.7–34.4; *p *< 0.001).

### CNS metastasis

At 1 year of diagnosis, 27% (95% CI, 23–30%) of patients developed brain metastasis, and at 2 years, 32% (95% CI, 21–43%). As treatment for brain metastasis, only 4 patients achieved criteria to undergo radiosurgery, none of the patients was submitted to brain surgery, and all other patients received whole brain irradiation. Independent associated factors comprised adenocarcinoma histological type (RR 5.2; 95% CI, 1.002–29; *p *= 0.0002) and CEA serum levels ≥ 40 ng/mL (RR 11.4; 95% CI, 1.7–74; *p *< 0.001) (Table 2). In the subgroup analysis of patients with adenocarcinoma histologycal type, frequency ± standard error of CNS metastasis development at 12 months of diagnosis were 16.4 ± 0.03% (95% CI, 16.34 – 16.46%) and 67 ± 0.09% (95% CI, 66.91 – 67.09), in patients with CEA serum levels < 40 ng/mL, and ≥ 40 ng/mL, respectively. And at 24 months of diagnosis, frequency ± standard error of CNS metastasis development were 20.2 ± 0.05% (95% CI, 20.19 – 20.21%) and 67 ± 0.09% (95% CI, 66.82 – 67.18%) in patients with CEA serum levels < 40 ng/mL and ≥ 40 ng/mL, respectively. EGFR tissue expression was not statistically significant in terms of CNS metastasis development.

**Table 2:** Associated factors with CNS metastasis development

N = 293	CNS Metastasis 12 months % (95% IC)	CNS Metastasis 24 months % (95% IC)	*p *Univariate Analysis	RR (95% CI)	*p *Multivariate Analysis
**Age**					
<60 years	20 (14.2–25.8)	36 (20.2–51.6)	0.79		
≥60 years	22 (16.1–27.8)	22 (16.1–27.8)			
**Gender**					
Female	21 (13.6–27.3)	31 (12.9–48.1)	0.86		
Male	22 (16.1–27.9)	33 (18.8–46.2)			
**Smoking History**					
Negative	24 (18.2–29.9)	36 (18.6–53.9)	0.22		
Positive	19 (13.1–24.8)	26 (12.3–39.7)			
**Histology**					
Adenocarcinoma	28 (22.1–33.8)	31 (23.1–38.8)	0.0002	5.2 (1.002–29)	0.05
*Others	7.8 (3.9–11.7)	29 (3.5–54.4)			
**CEA ≥ 40 ng/mL**					
Negative	12 (8–15.9)	24 (11.8–35.3)	<0.001	11.4 (1.7–74)	0.01
Positive	61 (45.4–76.8)	61 (45.4–76.8)			
EGFR***					
Positive	57 (56.6–57.3)	57 (56.6–57.3)	0.2		
Negative	45 (44.6–45.3)	45 (44.6–45.3)			

The endpoint for univariate analysis was CNS metastasis at 24 months.

CNS: Central Nervous System.

RR (95% CI): Relative Risk (95% Confidence Interval).

*Others: Squamous, Giant Cells, Undifferentiated.

CEA: Carcinoembryonic Antigen.

***: Tissue expression was determined only in 85 different

primary tumor samples (see text).

### Overall survival

**Figure 1:**
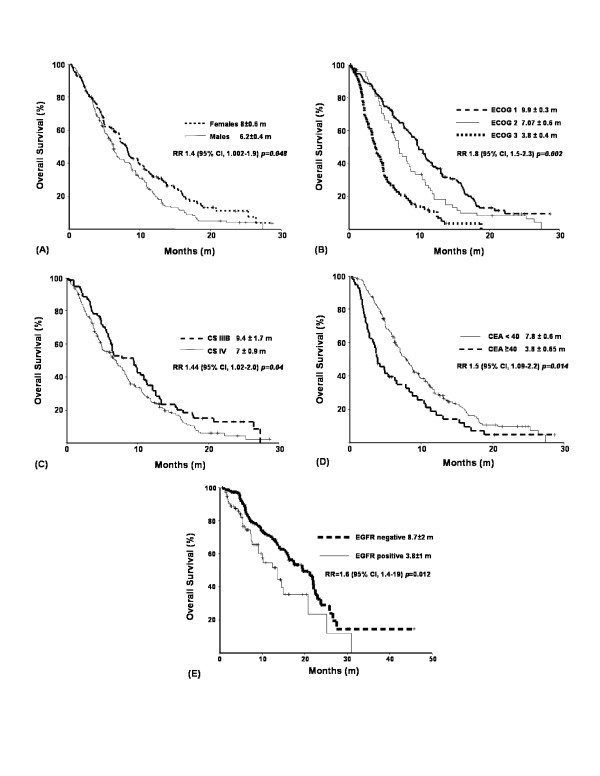
**Independent factors associated with overall survival**. RR (95% CI): Relative Risk (95% Confidence Interval). ECOG: Eastern Cooperative Ocology Group Scale. CS: Clinical Stage. CEA: Serum Carcinoembryonic Antigen Level. EGFR: Epidermal Growth-Factor Receptor.

OS was 7 ± 0.48 months. Factors associated with statistical significance at univariate analysis with poor OS were male gender (*p *= 0.02), age ≥ 60 years (*p *= 0.028), poor performance status (ECOG III, *p *< 0.001), serum CEA levels ≥ 40 ng/mL (*p *= 0.002), and EGFR-positive tissue expression (*p *= 0.023). Clinical stage IV demonstrated solely a tendency toward significance (*p *= 0.056) (Table 3). On multivariate analysis, male gender (RR = 1.4; 95% CI, 1.002–1.9; *p *= 0.048) [Fig 1a], poor performance status (RR 1.8; 95% CI, 1.5–2.3; *p *= 0.002) [Fig 1b], clinical stage IV (RR 1.4; 95% CI, 1.02–2; *p *= 0.04) [Fig 1c], serum CEA levels ≥ 40 mg/mL (RR 1.5; 95% CI, 1.09–2.2; *p *= 0.014) [Fig 1d], and EGFR tissue expression (RR 1.6; 95% CI, 1.4–19; *p *= 0.012) [Fig 1e] were statistically significant.

**Table 3:** Associated factors with overall survival

N = 293	Mean ± SE (months)	*p *Univariate Analysis	RR (95% CI)	*p *Multivariate Analysis
**Age**				
<60 years	8.33 ± 0.7	0.028	1.09 (0.8–1.47)	0.53
≥60 years	6.03 ± 0.7			
**Gender**				
Female	8.03 ± 0.57	0.02	1.4 (1.002–1.9)	0.048
Male	6.2 ± 0.41			
**Smoking History**				
Negative	7.2 ± 0.8	0.14		
Positive	6.4 ± 0.6			
**ECOG**				
1	9.9 ± 0.3	<0.001	1.8 (1.5–2.3)	0.002
2	7.07 ± 0.59			
3	3.83 ± 0.4			
**Clinical Stage**				
III B	9.4 ± 1.76	0.056	1.44 (1.02–2)	0.04
IV	7 ± 0.86			
**Histology**				
Adenocarcinoma	6.2 ± 0.8	0.67		
*Others	7.8 ± 0.69			
**CNS Metastasis at diagnosis**				
Negative	7 ± 0.6	0.9		
Positive	4.7 ± 1			
**Liver Metastasis at diagnosis**				
Negative	7.03 ± 0.5	0.51		
Positive	5.13 ± 2.6			
**CEA ≥ 40 ng/mL**				
Negative	7.8 ± 0.6	0.002	1.5 (1.09–2.2)	0.014
Positive	3.87 ± 0.65			
EGFR***				
Positive	3.8 ± 1	0.023	1.6 (1.4–19)	0.012
Negative	8.7 ± 2			
HER2***				
Positive	4.9 ± 2	0.3		
Negative	4.2 ± 2			

SE: Standard Error.

ECOG: Eastern Cooperative Oncology Group Scale.

*Others: Squamous, Giant Cells, Undifferentiated.

CNS: Central Nervous System.

RR (95% CI): Relative Risk (95% Confidence Interval).

CEA: Carcinoembryonic Antigen.

EGFR: Epidermal Growth-Factor Receptor.

***: Tissue expression was determined only in 85 different

primary tumor samples (see text).

## Discussion

Carcinoembryonic antigen, a glycoprotein expressed during early fetal life, is the product of the CEACAM5-gen. Its expression is restricted to epithelial cells, and it is found more abundantly on apical surface of gastrointestinal epithelium, but can also be found in other mucosal epithelia, such as lung [9]. In this prospective study, we found abnormal serum CEA levels (≥ 10 ng/mL) in 43% and levels ≥ 40 ng/mL in 22% of patients with advanced NSCLC. However, 41% of patients with pathological diagnosis of adenocarcinoma had serum CEA levels ≥ 40 ng/mL. In our study, we found a significant association between high CEA serum levels and adenocarcinoma in comparison with other histological types. This observation is consistent with a Japanese study, which showed association between high CEA serum levels and primary lung adenocarcinoma in clinical stage I, compared with squamous type [33].

We found that high CEA serum levels and histological type adenocarcinoma were associated with brain metastasis at time of diagnosis. Furthermore, we found that a CEA serum level ≥ 40 ng/mL is the more important factor associated with CNS metastasis development during follow-up (Table 2), independent of clinical stage (IIIB or IV) and absence or presence of liver metastasis, suggesting that association between high CEA serum levels and brain metastasis development are not due to tumor charge but rather to a more invasive phenotype.

Tumors with high CEA expression could possess an increased capacity to develop brain metastasis, and this could be due to vascular-tumoral cell-cell adhesion processes. CEA is a member of the IgCAM superfamily and is involved in homo and heterotypic interactions with other closely related IgCAMs, which possess at least one immunoglobulin-like domain [11]. Furthermore, CEA is usually found at higher concentrations in cerebrospinal fluid (CSF) of patients with metastatic tumors to CNS [34]. There is strong evidence regarding the capacity of CEA to penetrate the blood-brain barrier, behaving in a similar manner to Igs due to their homologous molecular weights [35,36]. Thus, CEA-positive tumor cells could bond to brain vasculature, favoring CNS metastasis development, similar to leukocyte transendothelial arrest and migration through blood-brain barrier mediated by integrin-Ig adhesion interactions [37]; in addition, this could be explained by paraprotein-secreting cells, such as mononuclear cells with Ig-kappa and lambda light chains, which preferentially pass from peripheral blood to CSF [38]. CEA could represent a molecular target in patients with lung adenocarcinoma. CEA blockage or expression inhibition could slow or even prevent brain metastasis development in this group of patients. Target blockade of CEA with antibodies inhibits the cell migration, invasion, and adhesion *in vitro *and *in vivo *in several tumor cell lines [39]. Moreover, CEA serum levels could be a recognition tool of patients with high risk of metastasis development, who may benefit from CNS imaging tests prior to development of neurological symptoms. Other cell-adhesion-molecular markers associated to lymph-node metastasis, as chemokine receptors CCR7, CXCR3 and CCL21 [40,41], could be related to brain metastasis development, thus, studies about analysis of their association with brain metastasis development are justified.

We found that OS-associated factors were gender, functional state, clinical stage, serum CEA levels, and EGFR tissue expression. Masculine gender is associated with a higher mortality risk than feminine gender. The eastern cooperative oncology group (ECOG) analyzed 1,594 patients, obtaining median survival of 9.2 months for females and 7.3 months for males (*p *= 0.004) [42], and Visbal *et al*. in 2004 conducted a study of 4,618 patients, finding a mortality risk for males of 1.2 (95% CI, 1.11–1.3) compared with females [43]. These findings are similar to our results [Fig. (A)]; however, they remain unexplained, and recent studies report contradictory results [44,45]. Evidence oriented in favor of females (best survival and local control post-surgery) is based on a different etiologic possibility: best tolerance to chemotherapy and possibly an estrogen role in lung oncogenesis. EGFR tissue expression increased the mortality risk in our patients by 60% compared with those not expressing EGFR. As showed by meta-analysis in 2002, detection of EGFR tissue expression by IHC was associated with a worse prognosis (mortality, RR 1.13; 95% CI, 1–1.28) [46]. As previously reported, HER2 tissue expression had a low incidence in our population, and we found no association between HER2 expression and brain metastasis development or survival. Nonetheless, this may be due to low frequency; thus, we cannot conclude about its impact on brain metastasis development or survival.

It is convenient to mention that we found differences between CNS metastasis prensence at diagnosis and overall survial rates in our cohort patients, according to cut-off levels of CEA serum levels ≥ 10, 20 30 y 40 ng/mL; however, related differences to both variables were more notorious with a cut-off point of CEA serum level ≥ 40 ng/mL, thus we selected this value to perform our subsequent analyses.

In many neoplasms, high serum CEA levels have been previously described as a predictor of residual disease or tumor relapse in patients without normal-range serum levels after surgery [47]. We prospectively reported that patients with CEA serum levels ≥ 40 ng/mL had an increased mortality risk of 50% as an independent feature. In a retrospective study in 70 patients in early stages of NSCLC, those with high CEA serum levels demostrated an OS at 3 years of diagnosis of 0%, compared to 18% in patients with normal levels. In fact, Iwasaki *et al*. proposed a formula to evaluate mortality risk based on CEA serum levels, histological type, and presence of positive mediastinal lymph nodes [48]. High CEA serum levels may reflect micrometastatic disease, although we detect no differences of CEA serum levels between patients in IIIB and in IV clinical stage. This observation suggests that the prognostic role of high CEA serum levels could be not due to other factors aside from tumor charge. CEA comprises an important tumor marker associated with several physiopathological processes of tumorogenesis, such as immunological defense, cell adhesion, cell survival, and metastasis, and its expression is induced by hypoxia-inducible factor α (HIF-α), suggesting that CEA plays an important role as a microenvironmental factor during tumorogenesis and confers a worse prognosis [9,49,50].

## Conclusion

High serum CEA level at diagnosis is an independent prognostic factor of CNS metastasis development and survival in patients with NSCLC. Surface expression of CEA in tumor cells could be a mechanism of invasion to CNS through immunoglobulin-related transport in blood-brain barrier. CEA may represent a potential molecular target.

## Competing interests

The authors declare that they have no competing interests.

## Authors' contributions

DSP, RK, LM, DMP, PC, AA, EG and JDG participated in the design and follow-up of patients. AAS performed the analysis of tumor specimens. DSP helped to draft the manuscript and contributed to the statistical analysis. OA conceived of the study, and participated in its design and coordination, performed the statistical analysis and helped to draft the manuscript. All authors read and approved the final manuscript.

## Pre-publication history

The pre-publication history for this paper can be accessed here:

http://www.biomedcentral.com/1471-2407/9/119/prepub
